# Notes and News

**Published:** 1887-02-19

**Authors:** 


### Notes and News.

Collected and Communicated.

Sir A. K. Rollit, Bart., M.P., and Mr. Cowley Lambart, M.P., have joined the Committee of the Great Northern Central Hospital.

Mr. H. W. Allingham, F.R.C.S., has been appointed Surgeon to the Great Northern Central Hospital, *vice* Mr. G. H. Makins, resigned.

D. Walter N. Syers, M.D., M.R.C.S., has been appointed Assistant Physician and Registrar at the Hospital for Epilepsy and Paralysis, 32, Portland Terrace, Regent's Park.

Miss Manson's paper, published in another column, was read by Dr. Bedford Fenwick at the meeting of The Hospitals Association, on Wednesday evening last. Dr. G. W. Potter presided, and there was a very full attendance. Miss Manson, though suffering from illness, was able to be present, we are glad to say, and an instructive discussion was raised by the paper, which we hope to publish next week.

To the little Cottage Hospital at Ilfracombe a grand doll's-house, furnished in the most approved style of upholstery, has recently found its way, thanks to the thought and kindness of a local lady, Mrs. Burgess. The house, or rather, one ought to say, the mansion, will prove a source of much amusement to the little children who may have to sojourn at this institution.

The Holmesdale Cottage Hospital at Sevenoaks has just passed its thirteenth year, and an excellent record of work it has. Patients come to it from some thirty neighbouring parishes; last year there were fifty-eight patients under treatment, ten more than in the previous twelve months. A Samaritan Fund has recently been added to the hospital, and is proving a valuable boon to the poor. We should be glad to see Samaritan Funds more generally associated with our Cottage Hospitals.

A correspondent writes: "Some people have queer ideas about the payment of nurses. Our hospital provides a district nurse for daily work amongst the poor, quite free, of course, and another for private cases, on the usual terms; the latter, a fine, portly person, the former, small, with a peculiar walk. The other day a lady, who wanted a cheap attendant for an invalid friend, called at the hospital to make inquiries, saying that she had been told by her servant that there was *a little lame nurse* sent out for five shillings a week, but that more was charged for the other. She went away unsuited."

We learn that on the 10th and 24th of next month papers on "The Development of the Art of Nursing," and "The Elements of Medical and Surgical Nursing," respectively, will be read by Miss Ethel G. Manson, Matron and Superintendent of Nursing at St. Bartholomew's Hospital, at 37, Lowndes Square, the residence of Theresa, Countess of Shrewsbury; whilst on the 17th and 31st, papers on "The Nurse and the Sick-room," and "The Different Branches of the Nursing Profession," will be read by Miss Mollett, of the National Hospital, Queen Square. Admission to any of these lectures will be granted to holders of tickets, which can be obtained free on application to Lady Shrewsbury. The opportunity for acquiring information based upon practical experience seems exceptional, and we imagine these lectures will be largely attended by those engaged in the noble profession of nursing the sick.

The following Hospital Sunday fixture has been made: March 13th, Wolverhampton.

The following Hospital Saturday fixture has been made: February 26th, Wigan.

Mrs. Oliver, of the Frome Cottage Hospital, has sent us a cutting from the local weekly paper, which gives the following details concerning the institution. The annual meeting of donors and subscribers was held on the 25th January, Mr. Henry Cockey presiding. The report was presented by Mr. R. Payne, the hon. sec., and stated that, in 1886, 108 patients were under treatment in the hospital, the largest number yet known. The treasurer's balance-sheet showed a material reduction in expenditure, more particularly under the head of housekeeping expenses, for which great credit was given by the committee to the matron, to whose careful management this result is to be attributed. The total income for the year 1886 was £376 16*s.* 10*d.*, and the total expenditure £287 13*s.* 5*d.*, leaving a balance to carry forward of £89 3*s.* 5*d.*

Year by year the Derbyshire Hospital for Sick Children has been expanding its sphere of usefulness, and, by dint of zealous effort, its funds have been made to keep pace with the splendid work which the institution is accomplishing on behalf of the children of the poor. The hospital was founded nine years ago, the record of the first twelve months being thirty-seven cases treated, of which sixteen were reported as cured, eleven relieved, three died, and seven still under treatment. In 1886, there were 322 cases treated, of which 228 were cured, sixty-seven relieved, seven died, seven made out-patients, and twenty remained in the hospital at the end of the period covered by the report. The Derbyshire Children's Hospital is fortunate in possessing the help of a very large number of ladies who devote themselves unflinchingly to the work of collecting. Miss Cupis, the lady superintendent, not only gives her time and her services without emolument, but also contributes from her own private purse £80 a year to the support of the hospital. The institution is, happily, kept free from debt.

The following vacancies are announced this week, the amount of the salary being placed in each case after the name of the institution :—

**Honorary Medical Officers.**

Cardiff Infirmary. Ophthalmic Surgeon.
Stafford Infirmary. Visiting Physician, qualified.

**Resident Medical Officers.**

Birmingham General Hospital. Two House Surgeons, surgical qualifications.—No salaries.
Chelsea Hospital for Women. Three Clinical Assistants, qualified. Fee required, £5 5s.
Hemel Hempstead Infirmary. House Surgeon and Assistant Secretary, doubly qualified.—£100.
Preston Infirmary. Senior House Surgeon, qualified.—£100.
Royal Hospital for Diseases of the Chest, City Road. Junior House Physician.—£50.

**Non-resident Medical Officer.**

St. Asaph Union. One, qualified.—£83.

**Matrons.**

Dorchester Hospital.—£60.
Lowestoft Hospital.—£50.
Wakefield Asylum. Chief Female Officer.—£60.

**Trained Nurses.**

Belgrave Hospital for Children. One.
Bridgewater Infirmary, Charge Nurse, Men's ward.
Cheyne Hospital, Chelsea. Night Nurse.
Evesham Sanatorium. Fever Nurse and Manager.—£30.
Great Northern Central Hospital. Charge Nurse, Female ward.—£20.
Middlesbrough Fever Hospital. Charge Nurse.—£30.
Portsmouth Royal Hospital. Night Nurse, Male ward.—£25.
Seamen's Hospital, Greenwich. One.—£20.
Truro Nurses' Home. Several.

**Probationers.**

Bristol Infirmary. Two ladies. Fee, 10 guineas.
Middlesbrough Fever Hospital. One.—£11.

---

The management of the Queen's Hospital, Birmingham, have issued an appeal to their fellow-citizens for the subscription of £10,000, to establish a Jubilee Fund for the maintenance of that institution. The sum seems a large one; but it is stated that numerous alterations and improvements are imperatively required, and that the expenditure of at least £5,000 is necessary to render the hospital adequate to the needs of the enormous population by whom its aid is sought, whilst a like amount is wanted to place the institution upon a satisfactory financial footing. At the annual meeting of the supporters, which was held on the 7th inst., Lord Leigh presiding, Sir James Sawyer moved a resolution approving of the proposal, and commented on the severe pressure upon the resources of the Queen's Hospital by the extraordinary depression through which all classes of the community have recently been passing. During 1886 the number of patients attended to reached the large total of 28,264, and many of these ought to have been admitted as in-patients, had the accommodation allowed of its being done. Such, however, was not the case, and the speaker felt that it was impossible for the staff to do their duty by these poor people unless the wealthier inhabitants of Birmingham relaxed their parsimony and subscribed more generously to the needs of their indigent neighbours. We trust that so stirring an appeal, coming from one of so great weight and authority as Sir James Sawyer, will not be disregarded, but duly recognised and generously responded to by the citizens of Birmingham.

---

The 109th annual meeting of the Governors of the Newcastle-upon-Tyne Dispensary was held on the 4th inst. at that institution, which is situated in Nelson Street, under the presidency of Mr. W. H. Holmes. During the past year there has been an astonishing increase (no less than 2,722) in the number of casual patients, which was so large as to surpass the record of any previous year since the establishment of the charity. This increase is attributed in the report, which was read at the meeting by the Honorary Secretary, to the falling off in the attendance at the casual department of the Newcastle Infirmary, occasioned by the recent adoption of a paying system, threepence being required from each patient before treatment can be obtained. 6,201 patients had been admitted with, and 16,218 without, letters of recommendation; the total number treated during the whole period being 22,936. The chairman, in moving the adoption of the report, referred to the increase in casual cases, and we gather, reading between the lines of his speech, that some system of inquiry into the pecuniary circumstances and status of those seeking relief at the Dispensary will soon be set on foot. There is little doubt that much abuse exists in this direction, and we trust that some sure remedy will before long be adopted by most of our medical charities.

---

The *Pall Mall Gazette* states, according to a report read at a recent session of the Berlin Cremation Society, that the minimum cost of the incineration of a Berlin "subject" at Gotha amounts to 430 marks, exclusive of church fees, singing, bell-ringing, mourning coaches and urn. An urn may be deposited, free of expense, in the crematorium for twenty years, at the expiration of which term its safe-keeping is to be paid for, or the urn will be properly interred, unless otherwise disposed of by the relatives. If the incinerated remains are to be interred in a Gotha cemetery immediately after being cremated, such interment must be paid for like any other; they may, however, be taken away in a tin case by the family, if so desired. The quantity of coal required for an incineration, as included in the foregoing specification, is two tons and a half, at twenty marks each; if several incinerations take place in the same day, only one ton and a quarter are charged after the first.

---

An "Unmusical Correspondent" writes :—"May I be permitted be congratulate 'the powers that be' in the Worcester Infirmary on their thoughtful kindness in providing the means of recreation for the nurses within the Infirmary? They evidently consider that a hospital should be, as far as possible, the *home* of those who work in it, rather than the place of penal servitude some institutions are made to resemble. As to the danger of annoying the patients, I think that it would be very slight, even though the nurses' room should be close to the wards. As a rule, sick people, unless very ill or singularly irritable, derive great pleasure from music and singing; and at the worst, I don't think that all the piano-playing that nurses could perform would cause a tithe of the annoyance endured from the frequent and merciless ringing of bells considered necessary in most establishments."

THE following sums have been recently received by the various Medical Charities, the name of the donor or the source from which they were obtained being given in each instance after the name of the Institution :—

**Donations.**

Dental Hospital of London, Leicester Square. Miss Green, £10 10*s*. Mr. C. C. Gooch, £10 10*s*. Mr. Henry M. Buckler, £10 10*s*.
Hospital Sunday Fund. Mr. Ernest A. Hankey, £495.
National Hospital for Consumption, Ventnor. "M. E.," £32.
North London Hospital. Mr. H. Wood, £10. Miss Parkes, £10. Mr. C. H. de Soysa, Ceylon, £50.
Royal London Ophthalmic Hospital, Moorfields. Mr. C. H. de Soysa, Ceylon, £25
British Home for Incurables, Putney. Mr. T. F. Marson, 40 guineas.
Chelsea Hospital for Women, Fulham Road. Miss M. Froud, £10 10*s*.
Royal Alexandra Hospital for Sick Children, Brighton. Mr. G. S. Kempson, £20.
Royal United Hospital, Bath. Mr. A. Spry, £10 10*s*. Mr. E. H. Wiggett, £25. Rev. W. A. Duckworth, £10 10*s*.
Sussex County Hospital. "A Friend," per Mr. N. P. Blaker, £10.
Hospital for Epilepsy and Paralysis, Portland Terrace, Regent's Park. Hon. Dudley Fortescue, £21. Mrs. H. Knight, £21.

**Collections.**

West London Hospital, Hammersmith. Offertory at St. Barnabas, Addison Road, £17 5*s*. 10*d*.
Derbyshire General Infirmary. Offertory, St. Alkmunds, £20 7*s*.

**Entertainments.**

Dispensary and Cottage Hospital, Ross. Mr. Eustace Power's Concert, £12 10*s*.
Meath Hospital and County Dublin Infirmary. Miss Elsner's Concert, £27 5*s*. 6*d*.
Victoria Hospital for Sick Children, Dublin. £50.

**Legacies.**

Hospital for Diseases of the Throat, Golden Square. Miss M. A. Cox, £50.
Torbay Hospital. Mr. Forster, £500.
Hospital for Epilepsy and Paralysis, Portland Terrace, Regent's Park. Miss Cumming, £500.

**Subscriptions.**

Meath Hospital and County Dublin Infirmary. Mrs. Hugh Brown, £10.
Hospital for Epilepsy and Paralysis, Portland Terrace, Regent's Park. Mr. G. Sturge, £50; half-yearly subscription.

---

THE annual meeting of the subscribers to the Dewsbury District Infirmary was held on the 25th ult., when the report of the board of management was adopted. This stated that, for the first time since the new building was opened, the current income had more than sufficed to meet the expenditure of the year, and the contributions of the working-classes had increased by £141. The increase in the Hospital Saturday contributions was attributed to the more general adoption of the system of weekly collections which now prevails. There are forty-five beds ready for use, the cost of each being £52 14*s*. 4*d*., as against £52 14*s*. in 1885, and £64 17*s*. in 1884. The average cost of each in-patient was £5 18*s*. 9*d*., as against £6 8*s*. 4*d*. in 1885. It has been found necessary to build a laundry, a mortuary, and a small stable for the horses of the honorary medical staff, and these are now in process of construction. The estimated cost for them is £1,200, which the ladies of the district hope to raise by means of a bazaar.

---

FOR the last twenty-eight years the Devonshire Hospital at Buxton has been no inconsiderable factor in the useful career of the ancient Bath Charity of that well-known health-resort, and the extension of the hospital buildings some four or five years ago has greatly increased the advantages available by its means for rheumatic and other patients. The hospital now possesses large accommodation, and extensive private grounds for the use of the sufferers, and affords every desirable care and attendance, both medical and otherwise. During the year 1886, 2,449 in-patients were admitted, of whom no less than 2,325 were discharged as improved, and only 48 as no better; a record which amply testifies to the admirable curative powers of the Buxton mineral water in rheumatism and allied complaints, since out of the whole number just quoted only 354 cases were without some degree of rheumatic complication.

---

THE financial report of the Devonshire Hospital is not quite so satisfactory as the medical. It is stated that, under all heads, the total receipts during the past year were less than in 1885 by £276 7*s*. 9*d*., and this falling-off is attributed by the authorities to the long-continued general depression, which has so seriously affected all interests in this country. We trust that the revival which is now slowly beginning to be felt, will make ample atonement for all past shortcomings, not only to the Buxton, but to every charity in the country, in the course of the present year.

---

THE Convalescent Home at Llanfairfechan has been found too small to accommodate the number of persons who desire admission; and a proposal for a new building is now under consideration. The institution, originally intended for the benefit of the inhabitants of Walsall, was only opened eighteen months ago, but, under the able management of Miss Butler, it has become most popular, and admission is eagerly sought by invalids in the immediate neighbourhood. Mr. Luck has kindly promised a site (the market value of which is estimated at fully £700), and the Trustees of the Walsall Home have guaranteed £700 in aid of the movement. Miss Butler states that she has a sum of £150 in hand, so we hope to hear shortly that the new Home is in course of erection. It is estimated that the total cost will be about £3,000.

---

"MOTHER," dating from the College, Glasgow, encloses us an epistle from a little child, "Sister Dozie"—for thus the tiny maiden calls herself—descriptive of her Christmas visit to the infirmary. The quaint language, "Mother" tells us, has been learnt from a Highland nurse.

"DER MR. EDILA,—Pese Ise now doin' to zite to you 'bout de 'Firmasy (Hospital) on Tissmus Day; tause you did say zoo wanted to hab a letter arl 'bout de 'Firmasies. Ise went wif sisters to see de Tissmus Tee der; oh such a booty Tissmus Tee we see'd; I are a bid dirl now and Ise diden't kye at arl. Der were lots of dollies for de ickle sick chillens, such booty dollies; so pitty, so pitty. Der was one booty dolly wid a booty wed sattin fok on. Sisters telled me it was wed sattin. Ise wanted is so vely, vely much, tause mine dolly did det its hed arl boke off. Ise did tall out vely loud for de booty doll arf de pitty Tissmus Tee; arl de ickle sick chillens det pitty dollies arf de Tissmus Tee. Not pore Sister Dozie, she diden't det nuffin. Pese dats arl now. Mamy talls me sum times her ickle woman. Ise not ickle woman, ise SISTER DOZIE."

